# Microglia Exhibit a Unique Intact HIV Reservoir in Human Postmortem Brain Tissue

**DOI:** 10.3390/v17040467

**Published:** 2025-03-25

**Authors:** Marieke M. Nühn, Stephanie B. H. Gumbs, Pauline J. Schipper, Irene Drosou, Lavina Gharu, Ninée V. E. J. Buchholtz, Gijsje J. L. J. Snijders, Frederieke A. J. Gigase, Annemarie M. J. Wensing, Jori Symons, Lot D. de Witte, Monique Nijhuis

**Affiliations:** 1 Translational Virology, Department of Medical Microbiology, University Medical Center Utrecht, 3584 CX Utrecht, The Netherlands; m.m.nuhn-3@umcutrecht.nl (M.M.N.); stephanie.gumbs@cchmc.org (S.B.H.G.); p.j.schipper@umcutrecht.nl (P.J.S.); n.v.e.buchholtz@umcutrecht.nl (N.V.E.J.B.); j.symons@umcutrecht.nl (J.S.); 2 Department of Psychiatry, Icahn School of Medicine at Mount Sinai, New York, NY 10029, USAf.gigase@erasmusmc.nl (F.A.J.G.); lot.dewitte@radboudumc.nl (L.D.d.W.); 3 Translational Virology, Department of Global Health and Bioethics, Julius Center for Health Sciences and Primary Care, University Medical Center Utrecht, 3584 CX Utrecht, The Netherlands; a.m.j.wensing@umcutrecht.nl; 4 Department of Psychiatry, Radboud UMC, 6525 GA Nijmegen, The Netherlands; 5 Department of Human Genetics, Radboud UMC, 6525 GA Nijmegen, The Netherlands

**Keywords:** HIV, brain, CNS, replication-competent, cellular tropism, microglia

## Abstract

A proviral reservoir persists within the central nervous system (CNS) of people with HIV, but its characteristics remain poorly understood. Research has primarily focused on cerebrospinal fluid (CSF), as acquiring brain tissue is challenging. We examined size, cellular tropism, and infection-dynamics of the viral reservoir in post-mortem brain tissue from five individuals on and off antiretroviral therapy (ART) across three brain regions. Microglia-enriched fractions (CD11b^+^) were isolated and levels of intact proviral DNA were quantified (IPDA). Full-length envelope reporter viruses were generated and characterized in CD4^+^ T cells and monocyte-derived microglia. HIV DNA was observed in microglia-enriched fractions of all individuals, but intact proviruses were identified only in one ART-treated individual, representing 15% of the total proviruses. Phenotypic analyses of clones from this individual showed that 80% replicated efficiently in microglia and CD4^+^ T cells, while the remaining viruses replicated only in CD4^+^ T cells. No region-specific effects were observed. These results indicate a distinct HIV brain reservoir in microglia for all individuals, although intact proviruses were detected in only one. Given the unique immune environment of the CNS, the characteristics of microglia, and the challenges associated with targeting these cells, the CNS reservoir should be considered in cure strategies.

## 1. Introduction

Following HIV transmission, people with HIV (PWH) are subjected to a lifetime of antiretroviral therapy (ART) to continuously suppress viral replication and prevent viral rebound from hidden anatomical and cellular HIV reservoirs [1,2,3]. Any successful effort to eradicate these latently infected cells will require a thorough understanding of the characteristics, the size and infection-dynamics of these reservoirs.

The largest and best characterized viral reservoir are the resting CD4^+^ T cells. These cells have high expression of the CD4 receptor and the CCR5 and/or CXCR4 coreceptors. HIV strains that target CD4^+^ T cells are referred to as T-tropic viruses, relying on the high expression of the CD4 receptor [4]. While CD4^+^ T cells are the best-characterized viral reservoir, HIV can also infect other cell types throughout the body, which may serve as additional reservoirs [5]. For instance, HIV can evolve to infect cells with low density of CD4 on the cell surface, such as myeloid cells. These CCR5-expressing cells are susceptible to M-tropic viruses [4,6]. It is important to highlight that, unlike T-tropic viruses, these so-called M-tropic viruses can infect both myeloid and T cells. We, therefore, rather refer to them as M/T-tropic viruses.

Over the past decade, research has provided compelling evidence that the CNS functions as an anatomical reservoir for HIV infection, as it has been linked to neurocognitive impairments, resulting in HIV-associated neurological disorders (HAND) [7]. Additionally, HIV RNA and/or DNA have been detected in the brain tissue of PWH, primarily localized to infected CD68^+^ microglia/macrophages [8,9,10,11,12,13], which constitute up to 1–10% of all brain cells [10]. Consequently, microglia are considered the predominant resident cellular reservoir of HIV within the brain [14]. Notably, one recent study identified a replication-competent clonal M/T tropic virus strain from postmortem brain tissue, although this finding was limited to a single case [15]. While several studies have indicated the presence of intact proviral DNA in brain tissue of PWH [11,16,17,18], this intact reservoir has not been specifically detected in microglia, and evidence for replication-competent virus in brain tissue remains limited.

The scarcity of brain tissue from people with HIV poses significant challenges in studying the cellular reservoirs of HIV in the brain. CD4^+^ T cells are present in low numbers within the CNS [19,20], raising uncertainty about their role as cellular reservoir in the brain of PWH. Both M/T-tropic and T-tropic viruses have been detected in the cerebrospinal fluid (CSF) of non-virally suppressed individuals [21,22,23]. The CSF, which serves as a barrier between the brain and the peripheral blood, contains predominantly T-tropic viruses as observed shortly after primary infection in virally unsuppressed individuals [21], during asymptomatic CSF escape in virally suppressed individuals [24], and as rebound viruses following treatment interruption [25]. Together, these findings suggest that, alongside microglia, T cells may also serve as a cellular reservoir in brain tissue, potentially contributing to rebound viremia. Further research focusing on the replication competence and cellular tropism of viruses within brain tissue, rather than CSF, is needed to substantiate these findings.

In this study, we used the intact proviral DNA assay (IPDA) [26,27] to examine the genomic integrity and quantify the proviral DNA within microglia isolated from three regions of postmortem brain tissue from five individuals with HIV. In addition, we characterized the replication-competence and cellular tropism of the *env* gene from these proviruses using both CD4^+^ T cells and a microglial culture model system, monocyte-derived microglia (MDMi) [28,29]. These findings will provide crucial insights into the HIV reservoir within the brain, which warrants careful consideration in HIV cure studies, as it may pose unique challenges for HIV cure compared to the peripheral blood reservoir [7].

## 2. Materials and Methods

### 2.1. Inclusion of Study Material 

Fresh post-mortem human brain tissue, ranging from 2 to 4 g, was collected from five donors within 48 h after death. The samples were obtained by the Manhattan HIV Brain Bank (MHBB; U24MH100931, 75N95023C00015), a member of the National NeuroAIDS Tissue Consortium, under protocol number R588. MHBB operates using protocols and consent documents approved by the Institutional Review Board (IRB) at the Icahn School of Medicine at Mount Sinai (ISMMS), New York City, NY, USA (the current project identification code is STUDY11-00388; the date of the current annual approval is 26 March 2024). The MHBB has been in continuous operation since 1999, using protocols in accordance with the Declaration of Helsinki. Written informed consent was obtained from the subjects or their primary next-of-kin before collecting and using autopsy tissues for medical research.

### 2.2. Human Brain Processing and DNA Isolation

Tissue was collected from all individuals from three different brain regions, namely the frontal lobe (FRONT), the subventricular zone (SVZ), and the occipital lobe (OCC). The tissue was mechanically and enzymatically dissociated to obtain a single cell suspension, followed by a Percoll gradient to remove myelin and red blood cells as previously described [30]. The enrichment of microglia was achieved using CD11b^+^ MACS (130-093-634, Miltenyi Biotec, Leiden, The Netherlands), according to the manufacturer’s protocol. DNA was isolated from the microglia-enriched (CD11b^+^) fraction using the DNeasy Blood & Tissue Kit (Qiagen, Venlo, The Netherlands) according to the manufacturer’s protocol.

### 2.3. Characterization of CD11b Microglia-Enriched Fraction

To characterize the CD11b microglia-enriched fraction, we used fresh postmortem brain tissue of a control donor provided by the Netherlands Brain Bank (NBB) (protocol 1233S3). In line with NBB policies, informed consent was provided by the donor ante mortem. Primary microglia were isolated as described above and characterized via flow cytometry. PBMCs from a healthy donor were isolated by Ficoll–Paque density gradient centrifugation of peripheral blood. Primary microglia and donor PBMCs were stained in cell-staining buffer (420201, Biolegend, Amsterdam, The Netherlands) with CD3-PE (SK7, BD), CD4-APC (SK3, BD), CD11b-PE-Cy7 (ICRF44, Fisher Thermo Scientific, Bleiswijk, The Netherlands) and CD45-FITC (2D1, Thermo Fisher Scientific) for 30 min. 7AAD (Thermo Fisher Scientific) was added 15 min before acquiring the samples. The samples were acquired on a FACSVerse (BD) and analyzed using FlowJo v10.

### 2.4. Droplet Digital PCR

The size of the proviral DNA reservoir was determined via the ultrasensitive quantification droplet digital PCR (ddPCR) method. The total proviral DNA reservoir was determined via the HIV-1 LTR ddPCR assay [31,32]. In this assay, samples with 0–1 positive droplets were classified as negative, those with 2–4 positive droplets were considered positive for proviral DNA and classified as containing “traces” of proviral DNA, and samples with 5 or more positive droplets could be quantified for their proviral DNA reservoir. The intact and defective proviral DNA was quantified using the IPDA ddPCR according to the previously described methods and cutoff values [27,32,33]. The IPDA assay targeted part of the *psi* and part of the *env* region [26,27]. Detection of both regions indicated the presence of intact proviruses, while detecting only the *psi* or *env* region signified defective proviruses. The *psi*-only and *env*-only copies accounted together for the total defective proviral DNA. Samples with a shearing index (DSI) of more than 50% and samples with a copy number below the limit of detection were excluded, as described previously [27]. Analysis of the results was performed with Quantasoft Version 1.7, for which replicate wells were combined before analysis. The graphs were generated with GraphPad Prism 10.

### 2.5. Generation of Recombinant Viral Clones

The HIV-1 envelope (gp160) gene was amplified using a 5-fold serial dilution to obtain single viral clones. Amplification was performed via a nested PCR (Platinum Taq Superfi PCR Master Mix, Thermo Fisher Scientific), according to the manufacturer’s protocol. Please refer to Table S1 for a list of the primers used. Envelope amplicons were introduced into a HxB2 gp160deletion vector with a luciferase reporter gene (HxB2ΔENVluc), previously described in [28], using the NEBuilder HiFi DNA Assembly Cloning Kit (New England Biolabs, Leiden, The Netherlands). We used the same vector carrying either the gp160 sequence of YU-2 (R5 M/T-tropic) or JRCSF (R5 T-tropic) (AIDS reagents) as controls. These plasmids were transfected into HEK-293 T cells (ATCC) using Lipofectamine 2000 reagent (Invitrogen, Thermo Fisher Scientific) to generate recombinant viral stocks. The supernatant containing replication-competent virus was harvested at 48 h post-transfection and 10× concentrated according to the manufacturer’s protocol (UFC903024, Merck Millipore, Amsterdam, The Netherlands) and stored at 80 °C until further use. p24 was determined with an ELISA p24 assay (DHP240B, Bio-Techne, Dublin, Ireland).

### 2.6. Sequencing

The recombinant viral clones were sequenced by Sanger sequencing (Macrogen, Europe). Overlapping primers were used to sequence the envelope (gp160). Primers are listed in Table S2. The sequences were edited using SeqScape and co-receptor tropism was predicted using Geno2Pheno v2.5 [34] with a >3.5% false-positive rate (FPR) cut-off. The nucleotide sequences were aligned using ClustalW in MEGA X v10.0.1 and a maximum likelihood phylogenetic tree (sampling with Bootstrap value 500 and General Time Reversible model) was generated in MEGA X.

### 2.7. CD4^+^ T Cell Isolation and HIV Infection

CD4^+^ T cells were collected through negative selection with the CD4^+^ T Cell Isolation Kit (Miltenyi Biotec 130-096-533) according to the manufacturer’s protocol from freshly isolated PBMCs from a healthy donor. These CD4^+^ T cells were stimulated for 2 days in culture medium (RPMI 1640, Gibco Life Technologies, Thermo Fisher Scientific) with 10% Fetal Bovine Serum, 1% gentamycin (^+^/^+^) (Gibco Life Technologies), and 20 U/mL IL-2 (Invitrogen) supplemented with Phytohemagglutinin (5 µg/mL). CD4^+^ T cells were infected in Duplo with 50 ng (p24 Gag) virus per 100,000 cells for 3 h in Eppendorf’s placed on a tube rotator. Maraviroc was administered to CD4^+^ T cells 1 h before infection at a concentration of 100 nM. After viral incubation, the cells were washed 3 times with culture medium with 10% RPMI containing IL-2 and cultured in 96-well plates in culture medium for 14 days without medium refreshment.

### 2.8. MDMi Culture and HIV Infection

CD14^+^ monocytes were collected through positive selection with the CD14^+^ T Cell Isolation Kit (130-118-906, Miltenyi Biotec) according to the manufacturer’s protocol from freshly isolated PBMCs. CD14^+^ monocytes were seeded in Poly-L lysine (P4707, Sigma-Aldrich, Amsterdam, The Netherlands)-coated 96-wells plates and allowed to rest for 2 h at 37 °C in monocyte culture medium: RPMI 1640, (Gibco Life Technologies), 2 mM I-glutamine, 1% gentamycin (Gibco Life Technologies). Subsequently, these monocytes were differentiated towards monocyte-derived microglia (MDMi) as described previously with minor modifications [28,29]. After 2 h, the medium was refreshed with monocyte culture medium, including 25% astrocyte-conditioned medium (ACM) (SCC1811, Sanbio, Uden, The Netherlands). On the fourth and eight day in culture, the medium was replaced with monocyte culture medium including growth factors: 10 ng/mL M-CSF, 10 ng/mL GM-CSF, 20 ng/mL TGFb, 12.5 ng/mL IFNg and 100 ng/mL IL-34 (all from Miltenyi Biotec). From day 8 onwards, differentiation of monocytes into microglial-like cells became more visible with elongated and stretched morphology (Figure S1A,B) and showed increased expression of microglia markers P2RY12, TREM2 and HLA-DR compared to monocytes, as confirmed by quantitative PCR from a previously described method [28] (Figure S1C). Infection was performed on day 10 post differentiation. The medium was replaced with microglia maintenance medium: RPMI 1640 (^+^/^+^), IL-34 100 ng/mL. MDMi were infected in duplicates with 50 ng (p24 Gag) virus per 175,000 cells. Maraviroc was administered to MDMi 1 h before infection at a concentration of 100 nM. The virus was incubated for 3 h and washed 3 times afterwards. The cells were cultured in this medium for 14 days without media refreshment.

### 2.9. Luminescence

The supernatant of the CD4^+^ T cell and MDMi experiments was collected 2–3 times per week and luminescence was measured with the Nano-Glo^®^ Luciferase Assay System (Promega, Leiden, The Netherlands) according to the manufacturer’s protocol. To exclude background signal during analysis, the luminescence reading measured on the first day post infection and after washing away the excess virus was subtracted from the luminescence readings taken on subsequent days for each sample. The graphs were generated using GraphPad v10.4.1.

## 3. Results

### 3.1. Study Participants

Human postmortem brain tissue was obtained from five individuals with HIV, with clinical details provided in Table 1. Viral suppression status was assessed based on the timing of their last recorded viral load (VL) and their ART regiments. Participants 1 and 5 were assumed to be non-virally suppressed, while participants 3 and 4 were considered virally suppressed due to their intake of ART prior to death. Due to a disruption of ART shortly before death, it was unclear whether participant 2 was virally suppressed.

### 3.2. Characterization of the CD11b^+^ Microglia-Enriched Fraction

Microglia are recognized to be the most predominant cellular reservoir of HIV within the brain. To study this microglia-specific reservoir, we isolated cells enriched for microglia (CD11b^+^) from non-frozen postmortem brain tissue of HIV-negative controls. Cytometric analyses showed that the CD11b-positive MACS-sorted cells were 97% pure for the myeloid cell markers CD45 and CD11b (Figure S2A). It is known that microglia have intermediate expression of CD45 compared to other myeloid cells [35,36,37,38]. Accordingly, our CNS-derived cells exhibited intermediate expression of this marker relative to peripheral monocytes (CD3^−^, CD4^+^, CD45^+^, CD11b^+^) (Figure S2B). Consistent with previous (transcriptomic) studies [35,36,37,39], the CD11b-positive fraction is predominantly composed of primary microglia. A small subset (1%) of macrophages with high CD45 expression is likely to be present within these CD11b-positive MACS-sorted populations (Figure S2A,B), as observed within plots of previous studies [38]. Overall, these results confirm that the CD11b-positive fraction consists primarily of primary microglia, with a minor presence of macrophages. Furthermore, within the CD11b-postive sorted fraction, no expression of CD3 or CD4 was detected in the CD11b- and CD45-negative cells (3%), confirming the absence of T cells.

### 3.3. Detection of (Intact) HIV DNA Within Different Brain Regions

**Figure 1:**
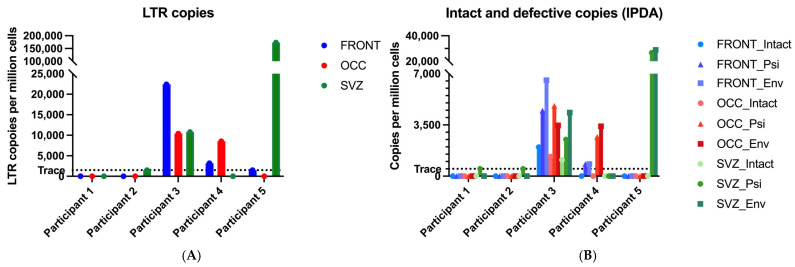
Quantification of the proviral reservoir. Copy numbers are indicated per million cells for all three brain regions in all five individuals. (**A**) The total proviral reservoir quantified via LTR copies. (**B**) The intact, *psi*, and *env* proviral copies are quantified with the IPDA. Single *psi* and *env* copies represent defective reservoirs. Traces of proviral DNA are defined as follows: for the LTR assay, reservoirs measured with 2–4 positive droplets, and for the IPDA, fewer than 7 detected copies, based on the limit of detection of the assay.

To determine whether the primary microglia from five PWH harbor a proviral HIV reservoir, we isolated primary microglia (CD11b^+^ cells) from three different brain regions: the frontal lobe (FRONT), the occipital lobe (OCC), and the subventricular zone (SVZ), Table 2. The total proviral DNA reservoir was determined based on the number of HIV LTR copies detected (Figure 1A and Table 2).

However, the total proviral LTR levels do not necessarily reflect the levels of intact proviral reservoir. Therefore, we also determined the levels of intact and defective proviral DNA separately in these individuals using the IPDA (Table 2). These results show that the dynamics of the total HIV DNA copies (LTR copies) closely correspond to the levels of defective proviral copies, as measured in the IPDA by detecting only a single *psi* or single *env* signal (Figure 1A,B). Participants 1 and 2 showed very low trace levels of single *psi* or *env* copies. For participants 3 and 4, the reservoir sizes, as measured in the IPDA, ranged from 823 to 6545 defective copies per million cells across brain regions, except for the absence of defective proviral copies in the SVZ of participant 4. Individual 5 displayed the largest variation in reservoir sizes between brain regions, with 26,645 and 28,892 copies per million cells in the SVZ for single *psi* and single *env*, respectively, to no proviral copies in OCC (Figure 1B). Interestingly, in participant 3, both intact and defective proviruses were detected, notably across all three brain regions (Figure 1B). The levels of defective and intact proviruses were comparable across brain regions for this individual. The size of the intact proviral reservoir ranged between 1111 and 1985 copies per million microglia, representing 13–15% of the total proviruses. In contrast, only defective proviruses were detected in the other four participants. In summary, HIV proviruses were detected in the brains of all participants across multiple brain regions, with an intact reservoir detected in one individual, underscoring the variability and complexity of CNS reservoirs.

### 3.4. Proviral DNA in the Brain Encodes Replication-Competent Envelopes

Following the detection of intact proviral DNA in participant 3, we opted to characterize the proviruses from these regions. To gain deeper genetic and functional insight into these proviruses, we amplified the full-length *env* gene from the viral DNA of microglia-enriched fractions of participant 3 across all brain regions. The *env* genes were inserted into a HXB2ΔENV luciferase vector to generate replication competent reporter viruses. We generated five clones per brain region (Table 3) to analyze the genetic and functional properties of the *env* genes. Genetic sequencing of the envelope showed that all clones were unique and predicted to be R5-tropic by Geno2Pheno, with False Positive Rates above 3.5 (Table 3).

**Figure 2:**
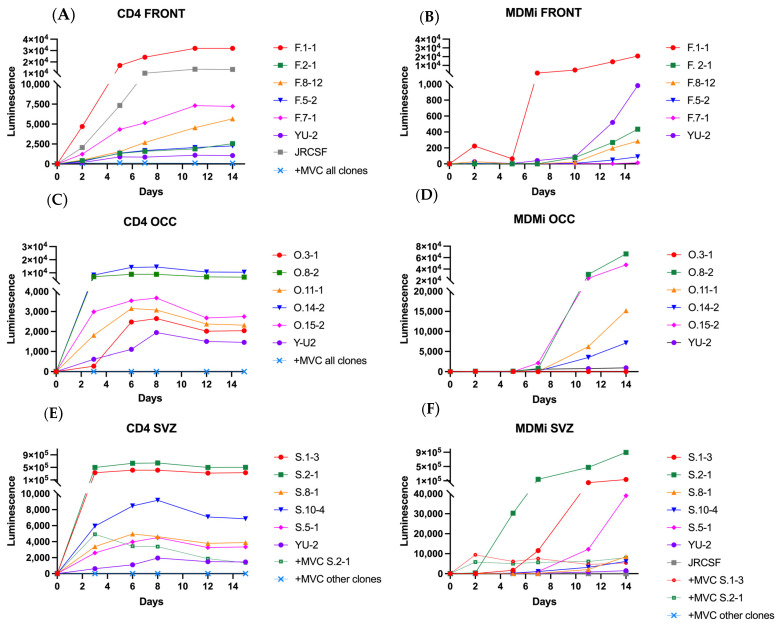
Infection dynamics of viral clones of participant 3. These viral clones of three different brain regions were used to infect CD4^+^ T cells (**A**,**C**,**E**) and MDMi (**B**,**D**,**F**) for 14 days. The *Y*-axis values are comparable but not identical between model systems and brain regions. All viruses were also monitored in the absence and presence of Maraviroc, a CCR5 inhibitor, prior to infection. Some viruses can replicate in MDMi, whereas all viral replications were inhibited by Maraviroc. MVC = Maraviroc.

Next, we assessed the capacity of these clones to produce replication-competent virus and to infect CD4^+^ T cells and microglia. All clones demonstrated the ability to productively infect and replicate within CD4^+^ T cells for up to 14 days post-infection (Figure 2). Clones from all brain regions showed varying luciferase levels, consistently producing higher viral loads compared to the control M/T-tropic strain YU-2 and comparable or higher as the T-tropic JRCSF control strain. Viral production was detectable as early as 2–3 days post-infection for all viruses. Furthermore, this replication was greatly inhibited by Maraviroc (MVC), a CCR5 inhibitor, indicating that these viruses are dependent on CCR5 (R5-Tropic) for viral entry. Only clone S.2-1 still showed low levels of viral production in presence of MVC, although its viral production without MVC was extremely high, suggesting insufficient MVC concentrations to fully inhibit infection.

In MDMi, viral production was observed in most clones, although with delayed dynamics by several days compared to CD4^+^ T cells (Figure 2). These infection patterns were generally consistent across duplicate experiments using cells from other donors (Figure S3). However, clones O.3-1, F.5-2, and F.7-1 consistently exhibited minimal to no viral production in both duplicate experiments, while clones O.11-1 and O.14-2 showed similar results in only one experiment, relative to the control strain YU-2. Clones that demonstrated high levels of viral production in MDMi across both duplicate experiments (F1.1, O-8.2, S.1-3, S.2-1) also showed high viral production in CD4^+^ T cells. In MDMi, viral replication was strongly inhibited by MVC, although some viral production persisted again in the high-producing clones S.1-3 and S.2-1 (Figure S3 and Figure 2). As expected, the T-tropic JRCSF viral strain only showed infection in CD4^+^ T cells, whereas the M/T-tropic YU2 infected both cell-types. These results indicate that most clones isolated from the three brain regions of participant 3 are R5 M/T-tropic, while three clones were restricted to infecting CD4^+^ T cells, identifying them as R5 T-tropic (Table 3).

**Figure 3:**
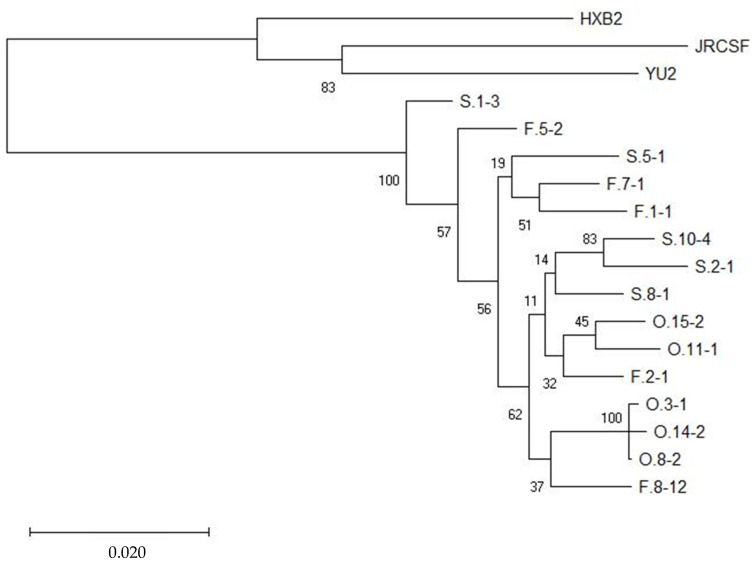
Maximum likelihood phylogenetic tree of envelope sequences of participant 3. The bootstrap values are shown next to each branch and a value of >70% support clustering. Created with MEGA X v10.0.1.

Interestingly, the generated maximum likelihood phylogenetic tree did not demonstrate a clear clustering of clones from a particular brain-region or cellular tropism (Figure 3), suggesting that neither factor is a primary driver of their genetic diversity. Together, these findings emphasize the heterogenous nature and diverse cellular tropism of the HIV reservoir across multiple brain tissue regions, underscoring the complexity of viral dynamics in the CNS.

## 4. Discussion

This study characterized proviruses in brain tissue of PWH, providing critical insights into the role of CNS reservoirs in HIV persistence. Proviruses were detected in microglia of the frontal lobe, the occipital lobe, and the subventricular zone in all individuals, including two who were on long-term ART. Intact proviral DNA was identified in all three brain regions of one ART-treated participant. Phenotypic characterization of the *env* gene revealed that the proviruses were R5-tropic and capable of infecting both MDMi and CD4^+^ T cells, although three of the 15 clones could only infect CD4^+^ T cells. These findings suggest that both R5 M/T- and T-tropic replication-competent proviruses in the brain could drive rebound viremia, underscoring the importance of targeting CNS reservoirs in HIV cure strategies.

Recent research increasingly suggests that the CNS may function as an anatomical reservoir for HIV [7,14,40]. For the CNS to function as a viral reservoir, HIV DNA would need to be present in resident cells capable of producing replication-competent viruses [7,14,41]. Multiple studies support this by showing intact proviral DNA in the brain [11,16,17,18], the presence of compartmentalized viruses in the CSF and brain tissue [21,22,23,42], and viral transcripts in the brain [13]. While the exact cellular source of this intact reservoir or replication-competent viruses remains uncertain, microglia are widely regarded as the primary source of independent viral replication in the CNS [14,15]. The most compelling evidence for this was recently shown by isolating clonal M/T-tropic replication-competent virus from microglia extracted from the brain tissue of a single individual with HIV [15]. Within our study, we further confirmed that microglia serve as a cellular reservoir for HIV, with HIV provirus detected in microglia isolated from the brain tissue of all five individuals examined. Notably, we identified an intact proviral reservoir in microglia from one ART-treated individual. Approximately 80% of the viral envelopes isolated from microglia tested from this individual were capable of replicating in MDMi, strongly suggesting that microglia are the cellular source of these replication-competent envelopes. While T cells infected with M/T-tropic viruses might also be engulfed by microglia [43], their isolation from microglia and ability to replicate within them indicate that microglia may serve as a cellular reservoir. The size of this intact viral reservoir ranged from 1111 and 1985 copies per million microglia, which is about 5–10 times higher than the previously reported 10 copies per million brain cells [17,18], given that microglia make up around 5% of the total brain cells [44]. Moreover, the intact reservoir compromised 15% of the total viral reservoir, compared to 8% and 11% in previous studies [11,17], a proportion larger than viral reservoirs found in the periphery after years of ART [33,45,46]. Interestingly, a recent study in 60 individuals found that total proviral reservoir levels were comparable across multiple tissues, including the brain, in virally suppressed versus virally unsuppressed individuals. However, in lymph nodes and the spleen, these levels were lower in virally unsuppressed individuals [47]. Similar findings have been reported in other studies specifically examining the brain [11,17]. This may be attributed to the immune-privileged status of the brain [48], which can result in reduced immune surveillance and limited cell-mediated killing. Furthermore, myeloid cells, such as microglia, have been shown to be more resistant to cell-killing compared to T cells [49]. Together, these factors likely contribute to the slower decline of the viral reservoir in the brain during ART, in contrast to the lymph nodes and spleen, where infected T cells may die because of massive replication. Nevertheless, the distribution of infection in CD68^+^ myeloid cells in the brain is uneven [12], making it challenging to compare sizes of brain reservoirs when looking to single biopsies. Together, these findings support the CNS as an anatomical reservoir for HIV, with microglia serving as an important cellular reservoir for independent viral replication. Further research in virally suppressed individuals, focusing on both proviral reservoirs and viral transcription, is essential to confirm whether and how viral latency persists within this compartment, a defining feature of anatomical reservoirs [7,14,41].

The brain is known for its complexity and heterogenous nature across different brain regions [50]. To explore this further, we compared the viral reservoir in three distinct brain regions: the frontal lobe, the occipital lobe and the subventricular zone. The microglia in the SVZ have been shown to be transcriptionally different compared to the other two more cortical brain regions [51]. Despite this, we did not find a consistent pattern of higher or lower number of infected cells within the SVZ as compared to the other regions. Interestingly, previous studies have shown varying results: one study showed higher levels of HIV in frontal white matter as compared to basal ganglia and cerebellum [17], whereas another reported higher levels of HIV in the frontal cortex and basal ganglia as compared to the occipital lobe [52]. Although these results might suggest that white matter regions in the frontal lobe harbor a larger HIV reservoir, direct comparisons with our study are challenging due to differences in biopsy methods and the precise regions targeted in each study. Furthermore, we did not find genetic clustering of viruses by brain regions, suggesting limited viral exchange between brain regions can occur as described before [53], even though compartmentalization between brain regions has been previously suggested [42,54]. These combined findings highlight the dynamic nature of the brain as an HIV compartment and the potential for infected cells to migrate between regions, contributing to the complexity of HIV reservoirs in the CNS.

Other cellular reservoirs, besides microglia, have also been suggested as potential reservoirs within the brain. Perivascular macrophages, along with other brain myeloid cells, have also been shown to harbor HIV proviral DNA [12,55]. In our characterization of the CD11b^+^ fraction from an HIV-negative control brain, approximately 1% of cells were identified as macrophages. While microglia appear to be the predominant reservoir, perivascular macrophages may also contribute to HIV persistence. Additionally, proviral DNA has been detected within a small subset of astrocytes [8,9,10], although the potential of astrocytes to be infected with HIV and to produce replication competent virus is still matter of debate. Some studies suggest entry of HIV in astrocytes via endocytosis or cell-to-cell mediated entry instead of CD4 receptor-mediated entry [56,57,58], although evidence for its ability to produce viruses remains limited [56,57,59]. Future research investigating HIV reservoirs and infection dynamics in isolated primary astrocytes from autopsy brains may help clarify these issues. Apart from microglia and astrocytes, the presence of T-tropic viruses in CSF and brain tissue suggests that T cells are also a cellular reservoir of HIV in brain tissue [21,22,23,24,25,60]. Interestingly, we identified both M/T- and T-tropic viruses in the microglia-enriched fraction from brain tissue. M/T-tropic viruses can infect both T cells and microglia, whereas T-tropic viruses are typically restricted to cells with high levels of CD4 expression [4,6]. Although T cells are typically rare in the brain, their numbers can increase during neuroinflammation [19,20]. As indicated in Table 1, there is no indication this is happening in participant 3. However, the detection of T-tropic virus in the microglia-enriched fraction suggests that (1) a limited numbers of T cells have been present, despite the 97% purity of this fraction for microglia and the absence of detectable CD3^+^ T cells, (2) T-tropic viruses may inefficiently infect microglia, or (3) microglia engulf infected CD4^+^ T cells or T-tropic viruses present in the brain [43]. Collectively, this suggests that both resident and infiltrating T cells might contribute to HIV persistence in the brain. Migrating infected T cells may facilitate the evolution of M/T-tropic strains capable of infecting microglia. This is supported by evidence that viral rebound strains within the CSF are predominantly T-tropic [24,25] and by the observation that T-tropic viral strains within the CSF are associated with an influx of T cells from peripheral blood and clonal proliferation, while M/T-tropic strains are genetically diverse and associated with established CNS infections [22]. Future in-depth sequencing in postmortem brain autopsies is needed to further study the role of T cells in the persistence of the HIV reservoir in the brain.

Furthermore, it is noteworthy to highlight that the characterization of an intact replication-competent HIV reservoir is subject to some well-known technical limitations. The IPDA primer/probe set excludes 97% of defective proviruses; however, up to 30% of intact proviruses may still harbor minor defects undetected by IPDA amplicons [26]. In addition, we were not able to study intact, full-length viruses isolated from the brains of people with HIV. Instead, we analyzed only the viral envelope derived from the total proviral DNA fraction. While infectivity and viral tropism are predominantly dependent on the viral envelope, we cannot exclude the possibility that potential defects outside of the envelope in, for example, Gag, Pol, Rev, Tat, or accessory proteins may impact viral replication, pathogenesis, or host–pathogen interactions [61,62]. Furthermore, although MDMi serves as a valuable model system to study CNS HIV infections, these cells are differentiated in vitro from peripheral blood monocytes. While they exhibit similarities to primary microglia [35,36,37,39], they do not fully replicate primary microglia. Therefore, despite significant advancements in PCR technology and CNS cell culture models, no technique or culture model is capable of accurately predicting the intact replication-competent reservoir. Thus, current estimates are likely to deviate from the true reservoir size and replication competence.

The presence of HIV proviruses in microglia from all five individuals, along with the detection of M/T- and T-tropic replication-competent proviruses in the microglia-enriched fraction from the brain of an ART-treated individual, reinforces the critical role of microglia as an important cellular reservoir for HIV in the CNS. This highlights the potential for CNS-derived viruses to drive viral rebound upon ART interruption. Given the immune-privileged environment of the CNS and the distinct responses of microglia to infection, reactivation, and cell-killing compared to peripheral cells [7], targeting HIV reservoirs in the brain poses unique challenges. These findings underscore the importance of carefully addressing CNS reservoirs in the design and implementation of effective and safe HIV cure strategies.

## Figures and Tables

**Table 1:** Overview of participant characteristics. VL: viral load. * Discontinued treatment 3 days prior to death. The second-to-last column indicates the number of days between the last CD4/VL measurement and death.

ID	Age	Sex	ART Regimen Prior to Death	Cause of Death	CD4 Count	VL Copies/mL in Plasma	Days to CD4/VL Pre-Death	Signs of T Cell Infiltration?
1	58	F	None	Acute subdural hematoma	228	2650	21 days	Leptomeningeal infiltrate
2	67	F	Abacavir, dolutegravir, lamivudine *	Bacterial sepsis	598	<20	124 days	No
3	65	M	Atazanavir, dolutegravir, tenofovir	Bacterial pneumonia	710	Undetectable	265 days	No
4	66	F	Emtricitabine/Rilpivirine/ Tenofovir Alafenamide	Hepatic necrosis	384	Undetectable	25 days	No
5	79	M	None	Bacterial pneumonia	1702	Undetectable	628 days	CD8^+^ T cell encephalitis

**Table 2:** Overview of HIV reservoir in CD11b^+^ fraction. The second column presents the DNA yield from the extracted CD11b^+^ fraction. The next columns include the number of HIV copies along with their corresponding measured cell counts in that assay. FRONT: frontal lobe, OCC: occipital lobe, SVZ: subventricular zone.

	CD11B^+^ Fraction	LTR		IPDA			
ID	DNA (ng)	Measured Cells	Copies/ Million Cells	Measured Cells	*Psi* Copies/Million Cells	*Env* Copies/Million Cells	Intact Copies/Million Cells
Pt. 1							
FRONT	4780	31,130	0	29,085	0	0	0
OCC	1020	7519	0	7989	0	0	0
SVZ	2920	15,829	0	28,196	Trace	0	0
Pt. 2							
FRONT	560	3366	0	2738	0	0	0
OCC	940	3586	0	5771	0	0	0
SVZ	2220	14,718	Trace	26,398	Trace	0	0
Pt. 3							
FRONT	7260	83,160	22,403	47,716	4470	6545	1985
OCC	3680	25,190	10,393	43,026	4795	3466	1341
SVZ	4840	33,660	10,784	31,124	2501	4339	1111
Pt. 4							
FRONT	960	5742	3180	9623	823	823	0
OCC	320	1727	8535	2457	2686	3403	0
SVZ	1120	2013	0	8404	0	0	0
Pt. 5							
FRONT	900	1859	Trace	6509	0	0	0
OCC	260	5170	0	7161	0	0	0
SVZ	800	286	173,077	685	26,645	28,892	0

**Table 3:** Recombinant *env*-clones of participant 3 and the control viruses and their characteristics. Co-receptor tropism was predicted based on false-positive rate (FPR) by Geno2Pheno after sequencing of the envelope (gp160), with an FPR > 3.5 considered as R5-tropic. The cellular tropism was assessed based on infection with the *env*-clones in CD4^+^ T cells or MDMi.

Brain Region	Clones	Predicted Coreceptor Tropism (FPR)	Cellular Tropism
FRONT	F.1-1	R5-tropic (3.7)	M/T-tropic
FRONT	F.2-1	R5-tropic (5.0)	M/T-tropic
FRONT	F.8-12	R5-tropic (4.7)	M/T-tropic
FRONT	F.5-2	R5-tropic (3.7)	T-tropic
FRONT	F.7-1	R5-tropic (3.7)	T-tropic
OCC	O.3-1	R5-tropic (3.7)	T-tropic
OCC	O.8-2	R5-tropic (3.7)	M/T-tropic
OCC	O.11-1	R5-tropic (5.0)	M/T-tropic
OCC	O.14-2	R5-tropic (3.7)	M/T-tropic
OCC	O.15-2	R5-tropic (5.0)	M/T-tropic
SVZ	S.1-3	R5-tropic (3.7)	M/T-tropic
SVZ	S.2-1	R5-tropic (3.8)	M/T-tropic
SVZ	S.8-1	R5-tropic (5.0)	M/T-tropic
SVZ	S.10-4	R5-tropic (5.0)	M/T-tropic
SVZ	S.5-1	R5-tropic (3.7)	M/T-tropic
Control	JRCSF	R5-tropic (31.7)	T-tropic
Control	YU2	R5-tropic (75.6)	M/T-tropic

## Data Availability

The data are available from the corresponding author upon request.

## Supplementary Materials

The following supporting information can be downloaded at https://www.mdpi.com/article/10.3390/v17040467/s1. Figure S1: Characterization of MDMi. (A) Morphological changes in MDMi following a 10-day differentiation of monocytes into MDMi, demonstrating an elongated and stretched phenotype. (B) Morphology of MDMi observed at 5 days post-infection in cells derived from the same donor. (C) Expression of microglia markers in monocytes and 10 days after differentiation of these monocytes into MDMi, highlighting the acquisition of microglial characteristics. Figure S2: Phenotype of the CD11b^+^ isolated fraction. (A) Phenotype of isolated primary microglia regarding their CD11b and CD45 expression and the presence of a small population of macrophages. (B) Comparison of isolated primary microglia, the isolated macrophages, and peripheral monocytes for their expression of CD45. Histogram plots are normalized to the mode of the set. Figure S3: Infection dynamics of duplicate experiments in MDMi. (A) Frontal lobe, (B) Occipital lobe, (C) Subventricular Zone. The Y-axis values are comparable but not identical brain regions. All viruses were also monitored in the absence and presence of Maraviroc, a CCR5 inhibitor, prior to infection. Some viruses are able to replicate in MDMi, whereas all viral replications were inhibited by Maraviroc. For comparable experiments, see Figure 2. MVC = Maraviroc; Table S1. PCR Primer sets for the amplification of the HIV *env* gene; Table S2. Primers used for Sanger sequencing of HIV-1 *env.*

## Abbreviations

The following abbreviations are used in this manuscript:

ART	antiretroviral therapy
CNS	central nervous system
CSF	cerebrospinal fluid
ddPCR	droplet digital PCR
DSI	shearing index
FPR	false-positive rate
FRONT	frontal lobe
HAND	HIV-associated neurological disorders
ISMMS	Icahn School of Medicine at Mount Sinai
IPDA	intact proviral DNA were quantified
IRB	institutional review board
MDMi	monocyte-derived microglia
MHBB	Manhattan HIV Brain Bank
MVC	maraviroc
NBB	Netherlands Brain Bank
NNTC	National NeuroAIDS Tissue Consortium
OCC	occipital lobe
PWH	people with HIV
SVZ	subventricular zone
VL	viral load
