# Supplementary material for: Microglia Exhibit a Unique Intact HIV Reservoir in Human Postmortem Brain Tissue
Source: Viruses. 2025 Mar 25;17(4):467. doi: 10.3390/v17040467 (PMC12030925; doi:10.3390/v17040467)
Supplement: Supplementary file 1 [file viruses-17-00467-s001.zip › Supplementary tables.pdf]

Supplementary tables

Table S1. PCR primer sets for the amplification of the HIV *env* gene.

HIV gene	PCR	Primers	Sequence 5'-3'
	First round	Oevif -1forw	GGGTCAGGGAGTCTCCATAGAATGGAG
		HIV-R-end-rev1	GCACACAACGCGTGAAGCACTCAAGGCAAGCTTTATTGAGGC
Env	Nested	gp160-fw-1	ACCAATAGTAGCAATAGTAGCATTAGTAGTAGCAGCAATAATAGCAATAGTTGTGTGG
		gp160-fw-2	ACCAATAGTAGCAATAGTAGCATTAGTAGTAGCAGCAATAATAGCAATAGTTGTATGG
		gp160-rv-3	GGAGTTCATGCTCAGCTCGTCTCATTCTTTCCCTTATAGCATGCCACCC
		gp160-rv-4	GGAGTTCATGCTCAGCTCGTCTCATTCTTTCCCTTATAGTAGGCCATCC

Table S2. Primers used for Sanger sequencing of HIV-1 *env*.

Forward primers	Sequence 5'-3'	Position in HXB2
LA45MOD	GCCTTAGGCATCTCCTATGGC	5954 - 5974
ENV-11	TGTGGGTCACAGTCTATTATG	6325 - 6345
ENV-14	CAAAGCCTAAAGCCATGTGT	6564 - 6583
V3-4	ACAGTACAATGTACACATGGAATTA	6954 - 6978
ENV-15	TTCTACTGTAATACAACA	7371 -7389
ENV-16	GGGTTCTTGGGAGCAGCAGG	7785 - 7804
ENV-17	GGCAAGTTTGTGGAATTGG	8224 - 8243
**Reverse primers**	**Sequence 5'-3'**	**Position in HXB2**
ENV-18	AAGCCTCCTACTATCATTA	8281 - 8299
ENV-21	ATGGGAGGGGCATACATTGC	7521 - 7541
ENV-22	TTCAGCTGTACTATTATG	7073 - 7090
ENV-23	GTGCTGATATTGAAAGAGCA	6693 - 6712
ENV-12	TGGGTTGGGGTCTGTGGGTA	6448 - 6467
