# Supplementary figures and images for: Microglia Exhibit a Unique Intact HIV Reservoir in Human Postmortem Brain Tissue
Source: Viruses. 2025 Mar 25;17(4):467. doi: 10.3390/v17040467 (PMC12030925; doi:10.3390/v17040467)

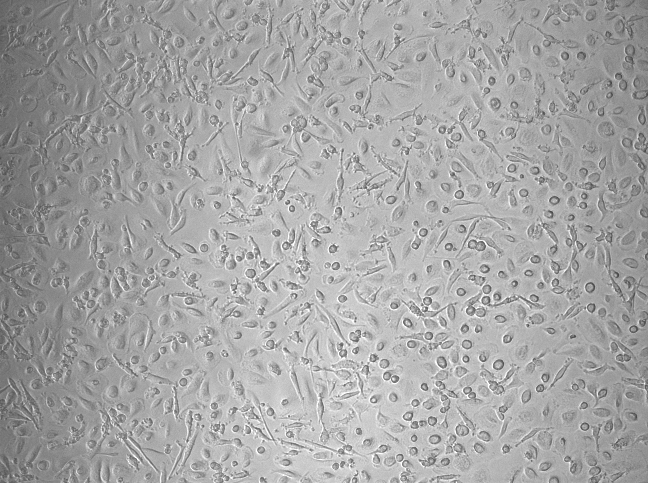

Supplement: Supplementary file 1 [file viruses-17-00467-s001.zip › Supplementary Figure S1A.jpg]

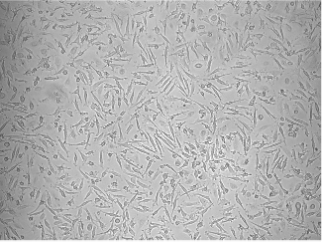

Supplement: Supplementary file 1 [file viruses-17-00467-s001.zip › Supplementary Figure S1B.png]

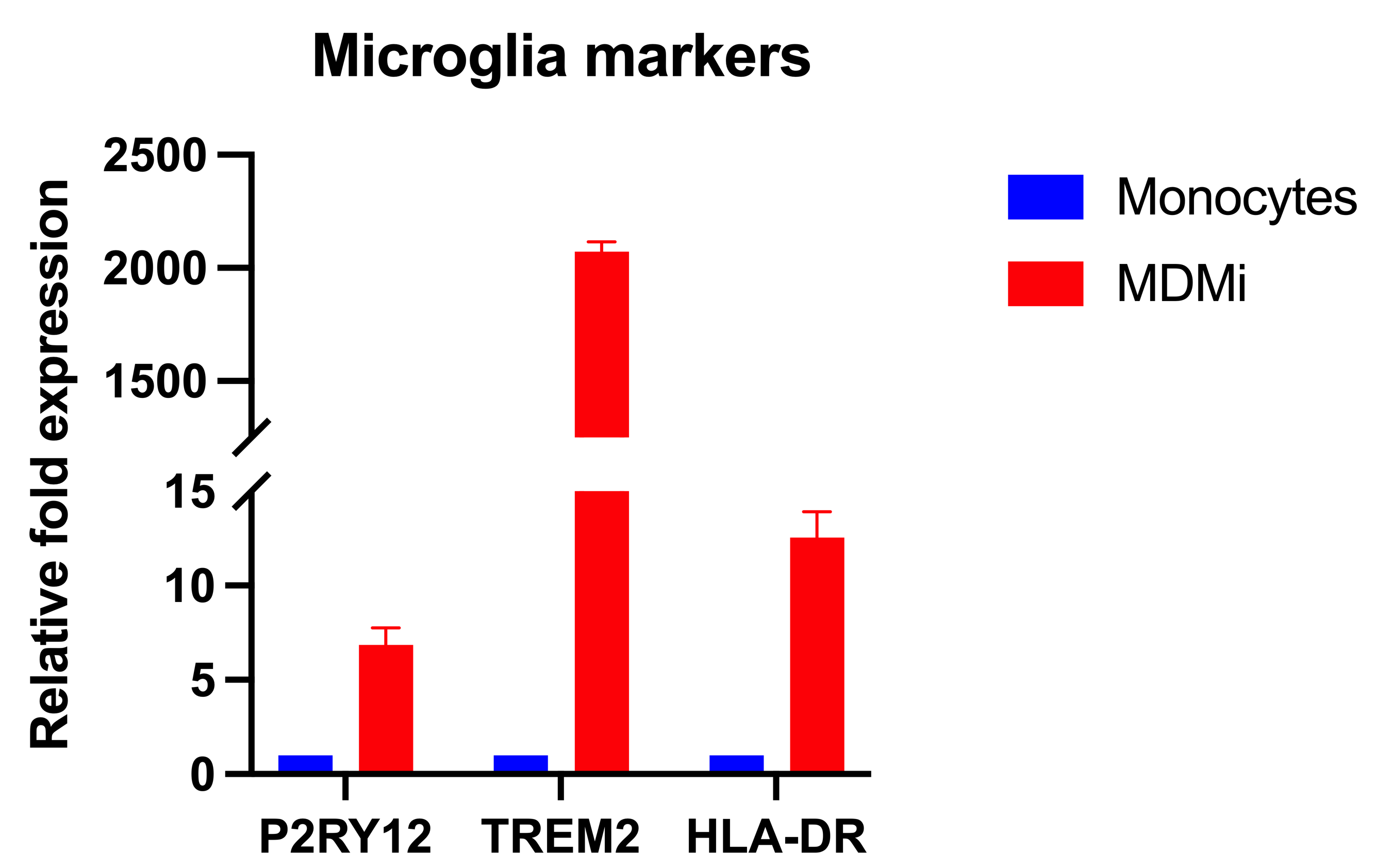

Supplement: Supplementary file 1 [file viruses-17-00467-s001.zip › Supplementary Figure S1C.png]

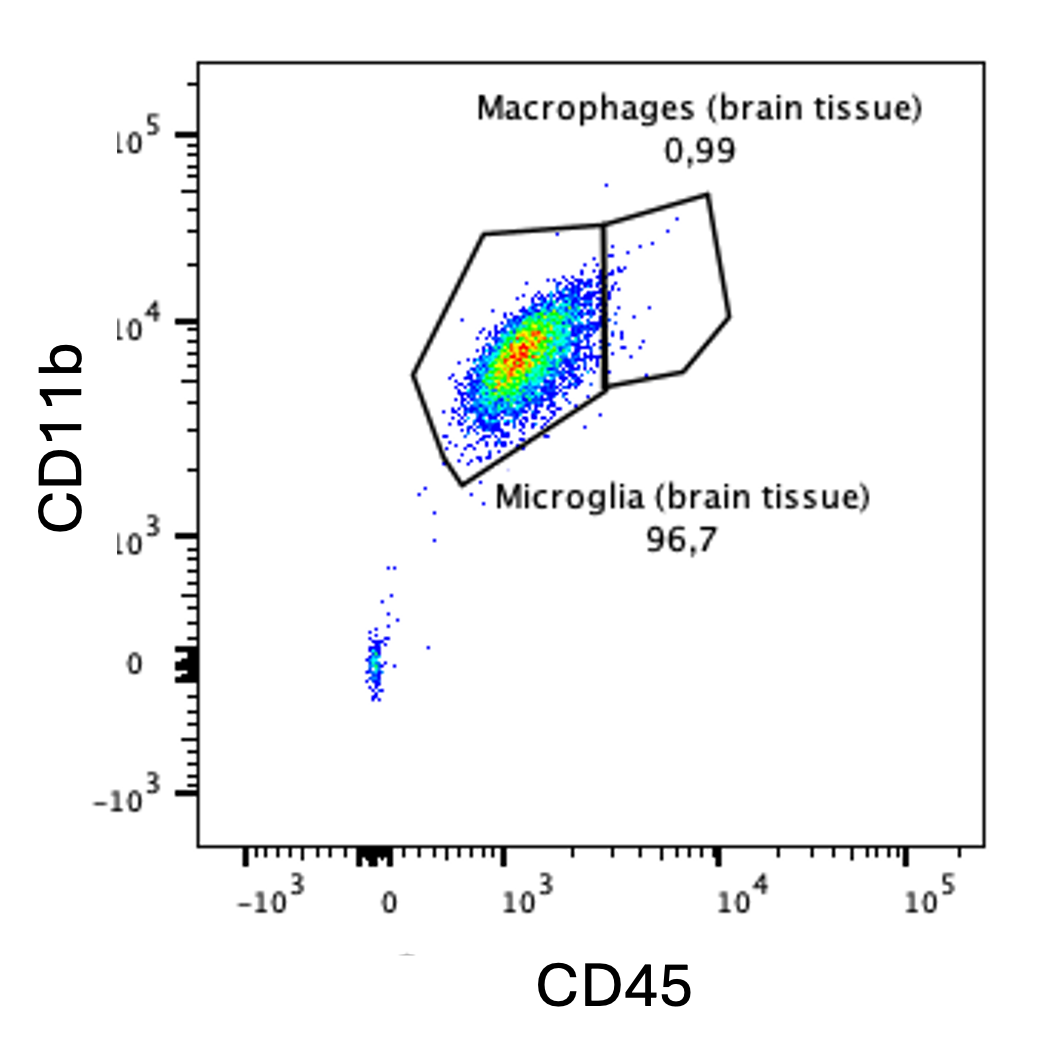

Supplement: Supplementary file 1 [file viruses-17-00467-s001.zip › Supplementary Figure S2A.png]

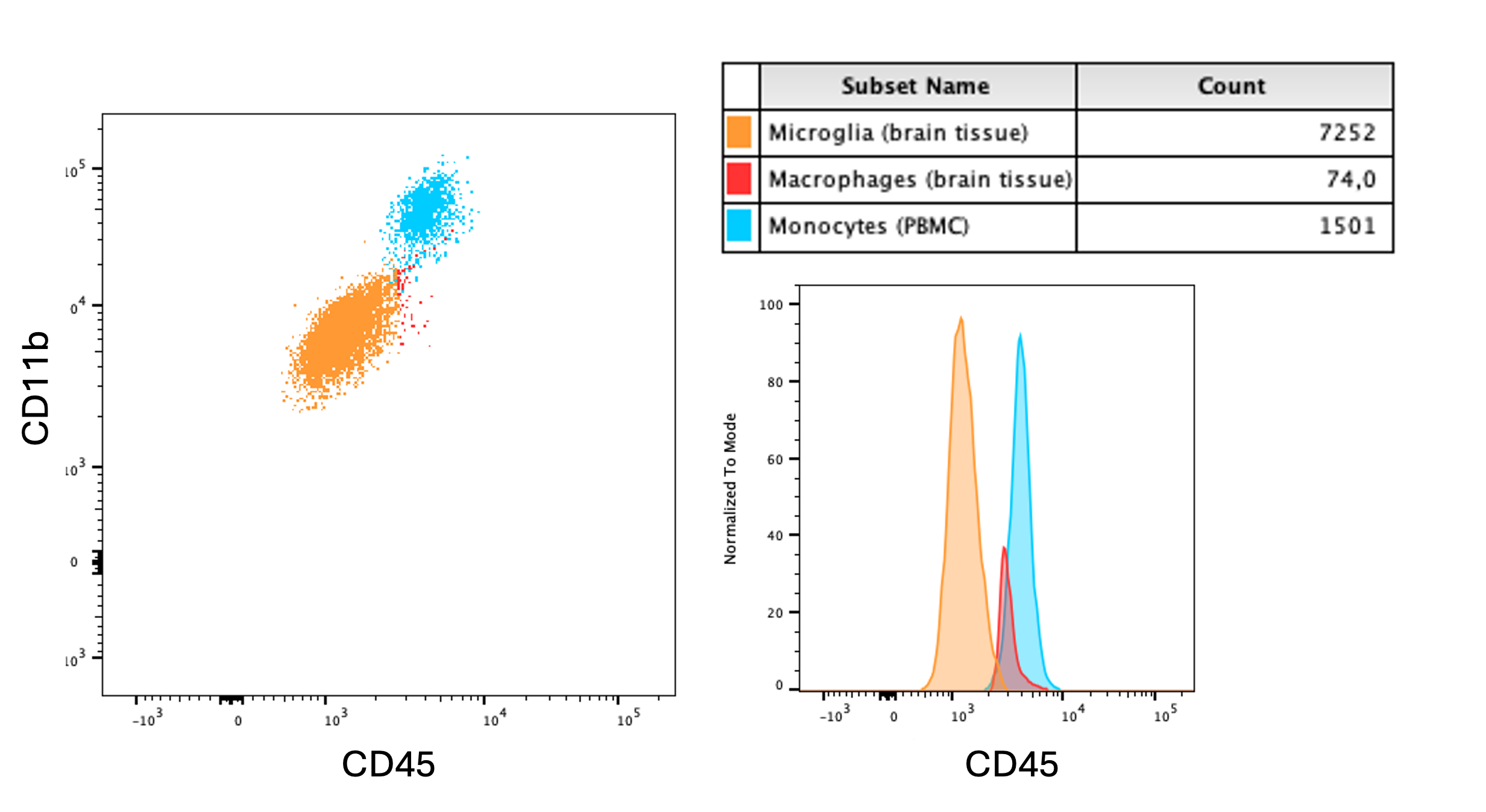

Supplement: Supplementary file 1 [file viruses-17-00467-s001.zip › Supplementary Figure S2B.png]

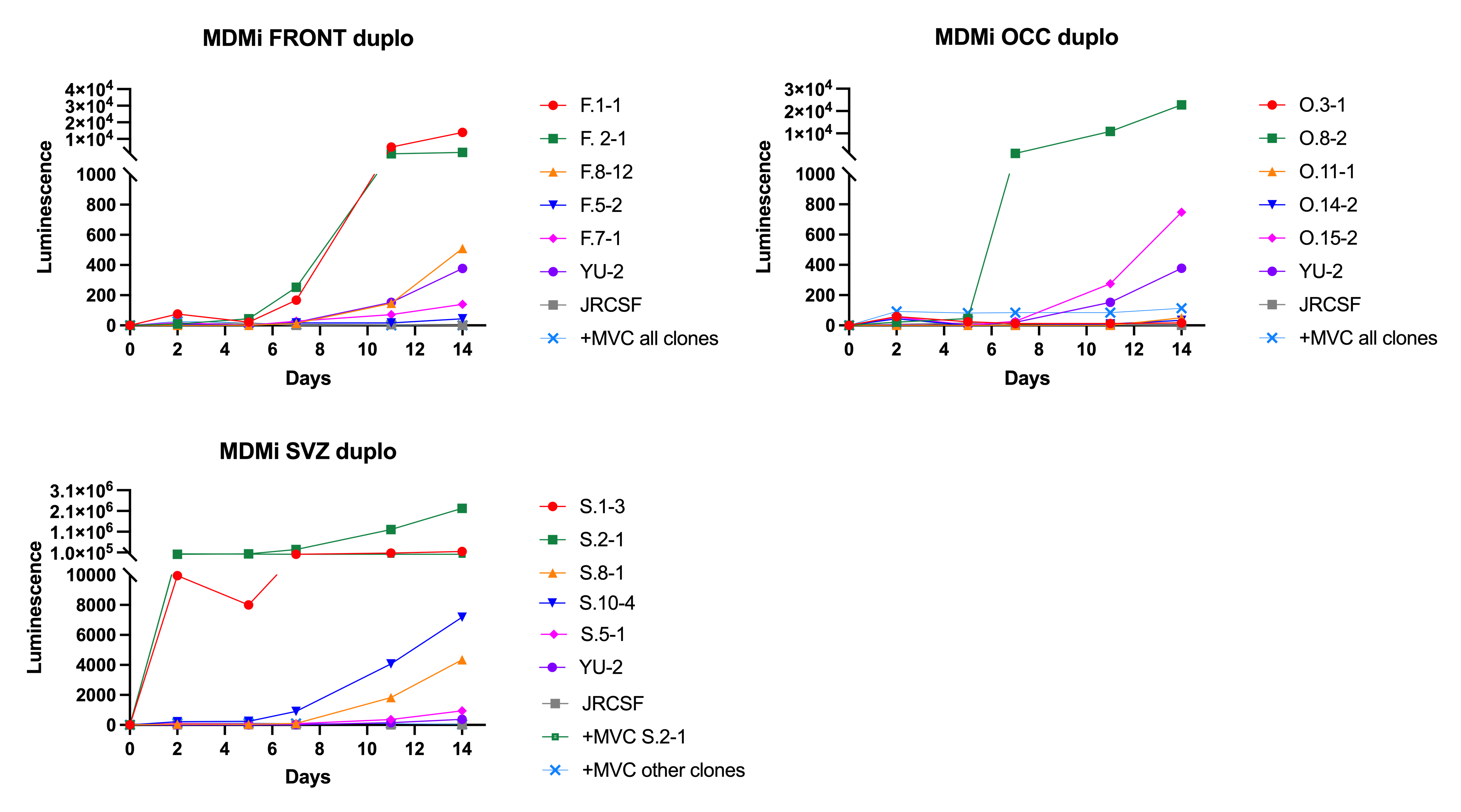

Supplement: Supplementary file 1 [file viruses-17-00467-s001.zip › Supplementary Figure S3.png]
